# Poly[[μ_3_-chlorido-bis(μ_2_-thio­urea-κ*S*)disilver(I)] nitrate]

**DOI:** 10.1107/S1600536810030953

**Published:** 2010-08-11

**Authors:** Saeed Ahmad, Aisha Saddiqa, Muhammad Monim-ul-Mehboob, Muhammad Altaf, Helen Stoeckli-Evans

**Affiliations:** a Department of Chemistry, University of Engineering and Technology, Lahore 54890, Pakistan; b Institute of Physics, University of Neuchâtel, rue Emile-Argand 11, CH-2009 Neuchâtel, Switzerland

## Abstract

The mol­ecular structure of the title polymeric complex, {[Ag_2_Cl(CH_4_N_2_S)_2_]NO_3_}_*n*_, consists of a binuclear cationic complex and a nitrate counter-ion. The cationic complex contains two bridging thio­urea (Tu) ligands and a triply bridging μ_3_-Cl anion. The latter is probably released from 2-amino­ethane­thiol hydro­chloride during the synthesis. The coordination environment around the two Ag^I^ atoms is different; one is trigonal planar, being coordinated by two thio­urea ligands through the S atoms and to one Cl^−^ ion, while in the other the Ag^I^ atom is tetra­hedrally coordinated by two thio­urea ligands through the S atoms and to two Cl^−^ ions. These units aggregate through the Cl^−^ anion and the Tu S atoms, forming a chain propagating in [100]. In the crystal structure, the polymeric chains are linked *via* N—H⋯O and N—H⋯Cl hydrogen bonds, forming a double layer two-dimensional network propagating in (011).

## Related literature

For silver(I) complexes with sulfur-containing ligands with applications in medicine and analytical chemistry, see: Raper (1996 ▶); Akrivos (2001 ▶). For silver(I) complexes containing thio­nes, see: Stocker *et al.* (2000 ▶); Pakawatchai *et al.* (1996 ▶); Casas *et al.* (1996 ▶); Aslandis *et al.* (2005 ▶); Ashraf *et al.* (2004 ▶); Isab *et al.* (2002 ▶). For silver(I) complexes containing thiol­ates, see: Nomiya *et al.* (2000 ▶); Zachariadis *et al.* (2003 ▶); Tsyba *et al.* (2003 ▶). For argentophilic inter­actions, see: Nomiya *et al.* (2000 ▶); Zachariadis *et al.* (2003 ▶); Tsyba *et al.* (2003 ▶). For the structures of some silver(I) complexes of thio­urea, see: Udupa *et al.* (1976 ▶); Hanif *et al.* (2007 ▶).
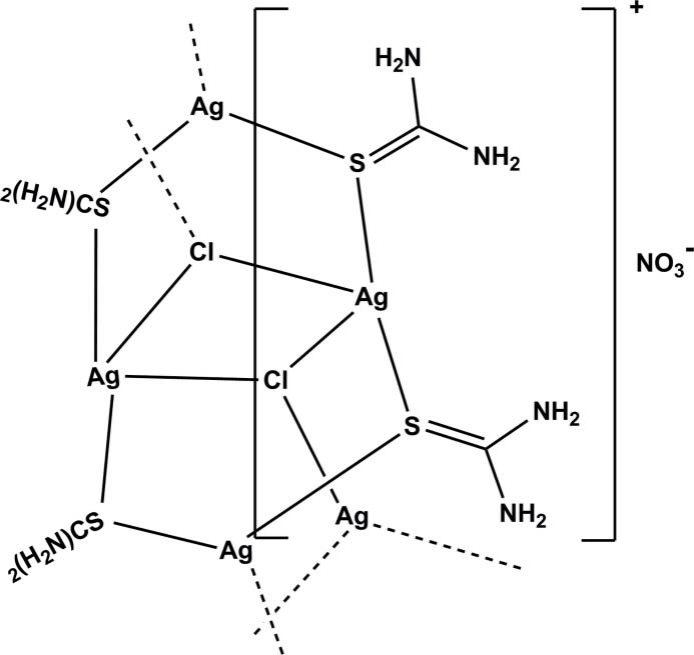


## Experimental

### 

#### Crystal data

[Ag_2_Cl(CH_4_N_2_S)_2_]NO_3_
*M* *_r_* = 465.44
Triclinic, 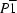

*a* = 6.3981 (8) Å
*b* = 7.7060 (9) Å
*c* = 11.8478 (14) Å
α = 83.041 (14)°
β = 82.868 (14)°
γ = 77.312 (14)°
*V* = 562.80 (12) Å^3^
*Z* = 2
Mo *K*α radiation
μ = 4.08 mm^−1^
*T* = 173 K
0.34 × 0.23 × 0.12 mm

#### Data collection

Stoe IPDS diffractometer
Absorption correction: multi-scan (*MULscanABS* in *PLATON*; Spek, 2009 ▶) *T*_min_ = 0.771, *T*_max_ = 1.353
4473 measured reflections
2055 independent reflections
1682 reflections with *I* > 2σ(*I*)
*R*_int_ = 0.056

#### Refinement

*R*[*F* ^2^ > 2σ(*F* ^2^)] = 0.044
*wR*(*F* ^2^) = 0.114
*S* = 0.98
2055 reflections
136 parameters
H-atom parameters constrained
Δρ_max_ = 1.49 e Å^−3^
Δρ_min_ = −1.27 e Å^−3^

### 

Data collection: *EXPOSE* (Stoe & Cie, 2004 ▶); cell refinement: *CELL* (Stoe & Cie, 2004 ▶); data reduction: *INTEGRATE* (Stoe & Cie, 2004 ▶); program(s) used to solve structure: *SHELXS97* (Sheldrick, 2008 ▶); program(s) used to refine structure: *SHELXL97* (Sheldrick, 2008 ▶); molecular graphics: *PLATON* (Spek, 2009 ▶) and *Mercury* (Macrae *et al.*, 2006 ▶); software used to prepare material for publication: *PLATON* and *SHELXL97*.

## Supplementary Material

Crystal structure: contains datablocks I, global. DOI: 10.1107/S1600536810030953/bt5309sup1.cif

Structure factors: contains datablocks I. DOI: 10.1107/S1600536810030953/bt5309Isup2.hkl

Additional supplementary materials:  crystallographic information; 3D view; checkCIF report

## Figures and Tables

**Table 1:** Hydrogen-bond geometry (Å, °)

*D*—H⋯*A*	*D*—H	H⋯*A*	*D*⋯*A*	*D*—H⋯*A*
N1—H1*A*⋯O3^i^	0.88	2.28	3.153 (8)	170
N2—H2*A*⋯O1^i^	0.88	1.95	2.831 (7)	177
N2—H2*B*⋯O3	0.88	2.11	2.932 (7)	155
N3—H3*A*⋯O1^ii^	0.88	2.00	2.881 (7)	174
N3—H3*B*⋯O2^iii^	0.88	2.08	2.930 (7)	163
N4—H4*A*⋯O2^ii^	0.88	2.22	3.095 (8)	173
N1—H1*B*⋯Cl1^iv^	0.88	2.56	3.372 (6)	155
N4—H4*B*⋯Cl1^v^	0.88	2.62	3.396 (6)	147

Symmetry codes: (i) 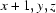; (ii) 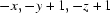; (iii) 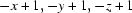; (iv) 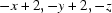; (v) 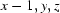.

**Fig. 1.**
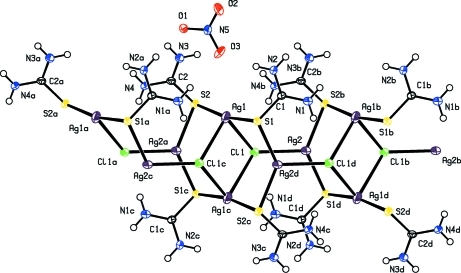
A view of the molecular structure of the title complex with displacement ellipoids drawn at the 50% proability level [Symmetry codes: (a) = -1 + x, y, z; (b) = 1 + x, y, z; (c) = 1 - x, 2 - y, 1 - z; (d) = 2 - x, 2 - y, -z].

**Fig. 2.**
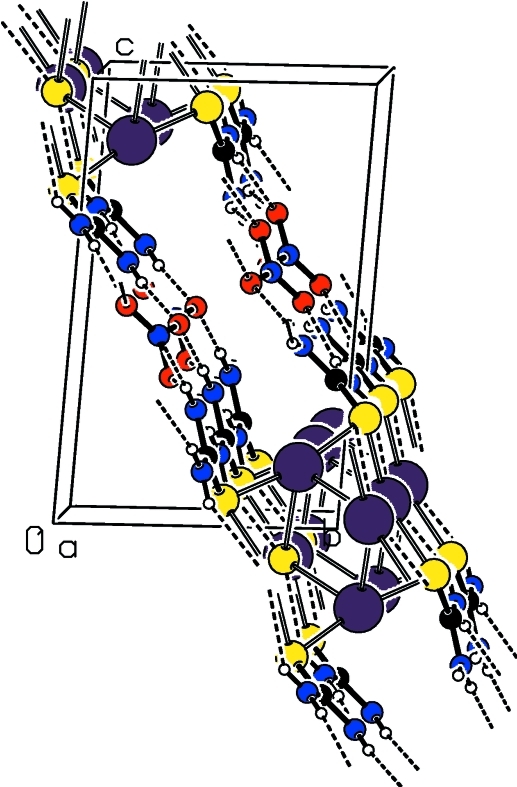
A view along the a-axis of the crystal packing of the title complex showing the N—H···O and N—H···Cl hydrogen bonds [dotted lines; see Table 1 for details].

## supplementary crystallographic information

### Comment

The study of the coordination and structural chemistry of silver(I) complexes with sulfur containing ligands has been a matter of interest over the last decades due to their wide range of applications in medicine and in analytical chemistry (Raper, 1996; Akrivos, 2001), and also due to their ability to adopt geometries with variable nuclearities and structural diversity. Consequently, several silver(I) complexes containing thiones (Stocker *et al.*, 2000; Pakawatchai *et al.*, 1996; Casas *et al.*, 1996; Aslandis *et al.*, 2005; Ashraf *et al.*, 2004; Isab *et al.*, 2002) and thiolates (Nomiya *et al.*, 2000; Zachariadis *et al.*, 2003; Tsyba *et al.*, 2003) have been prepared and structurally characterized. Silver(I) complexes with thiolates like thiomalic acid, thiosalisalic acid and 2-mercaptonicotinic acid [Nomiya *et al.*, 2000; Zachariadis *et al.*, 2003] also have remarkable antimicrobial activities for bacteria, yeast, and mold. The present report describes the structure of the new title silver(I) cluster of thiourea (Tu).

The molecular structure of the title complex is shown in Fig. 1. The asymmetric unit consists of a binuclear cationic complex and a nitrate counter ion. The cationic complex contains two silver(I) atoms, Ag1 and Ag2, two Tu ligands which bridge the silver(I) atoms *via* the S-atoms, and a triply bridging (µ_3_)Cl^-^ anion. The latter is probably released from 2-aminoethanethiol hydrochloride during the synthesis. The coordination environments around the two silver atoms are different. Atom Ag1 possesses a tetrahedral geometry, being coordinated to two thiourea ligands through the S-atoms, and two Cl^-^ anions. Atom Ag2 has a trigonal planar geometry, being coordinated to two thiourea ligands through S-atoms and to one Cl^-^ anion. These units aggregate through the Cl^-^ anion, and the Tu sulfur atoms, to form a one-dimensional chain which propagates in [100], as shown in Fig. 1.

The Ag—S distances around the trigonally coordinated Ag2 center [Ag2—S1 = 2.4827 (15) Å, Ag2—S2 = 2.4913 (16) Å] are somewhat longer than those around tetrahedrally coordinated Ag1 center [Ag1—S1 = 2.4305 (15) Å, Ag1—S2 = 2.4278 (15) Å]. In contrast the Ag—Cl bond distances are lengthened. The Ag1—Cl1 and Ag1—Cl1*^c^* [symmetry code: (*c*) 1 - *x*, 2 - *y*, 1 - *z*] distances are 2.8393 (15) and 2.9280 (16) Å, respectively, compared to distance Ag2—Cl1 which is 2.5477 (14) Å. The individual distances and angles within the Tu ligand are comparable to those reported for other Ag-thiourea complexes [Udupa *et al.*, 1976; Hanif *et al.*, 2007].

The shortest silver(I)···silver(I) distance of 3.2889 (8) Å [Ag1—Ag2*^a^*; symmetry code: (*a*) -1 + *x*, *y*, *z*] indicates that the complex is stabilized by significant argentophilic interactions. This distance is comparable to values reported previously [Nomiya *et al.*, 2000; Zachariadis *et al.*, 2003; Tsyba *et al.*, 2003]. The other short Ag···Ag distances include Ag2···Ag2*^d^* and Ag1···Ag2*^d^* of 3.5169 (8) and 3.5753 (8) Å, respectively [symmetry code: (*d*) 2 - *x*, 2 - *y*, -*z*], see Fig. 1.

In the crystal the polymeric chains are linked *via* N—H···O hydrogen bonds, involving the thiourea NH_2_ H-atoms and the nitrate O-atoms, and N—H···Cl contacts (Fig. 2, Table 1), to form a double layer two-dimensional network propagating in plane (011).

### Experimental

The title complex was prepared by adding 1 mmol (0.113 g) of 2-aminoethanethiol hydrochloride, dissolved in 10 ml of distilled water, to 1 mmol (0.170 g) of AgNO_3_, dissolved in 30 ml of distilled water. The mixture was stirred for 15–20 min giving a clear solution. 1 mmol (0.076 g) of thiourea, dissolved in 10 ml of methanol, was then added and the mixture was stirred for a further 15 min. The solution was then filtered and the filtrate kept at RT for slow evaporation of the solvent. After 2–3 days colourless crystals, suitable for X-ray diffraction analysis, were obtained.

### Refinement

The NH_2_ H-atoms could be located in difference electron-density maps. In the final cycles of least-squares refinement they were included in calculated positions and treated as riding atoms: N—H = 0.88 Å, with *U*_iso_(H) = 1.2*U*_eq_(N).

### Crystal data

**Table:**

[Ag_2_Cl(CH_4_N_2_S)_2_]NO_3_	*Z* = 2
*M**_r_* = 465.44	*F*(000) = 444
Triclinic, *P*1	*D*_x_ = 2.747 Mg m^−^^3^
Hall symbol: -P 1	Mo *K*α radiation, λ = 0.71073 Å
*a* = 6.3981 (8) Å	Cell parameters from 5310 reflections
*b* = 7.7060 (9) Å	θ = 2.7–26.0°
*c* = 11.8478 (14) Å	µ = 4.08 mm^−^^1^
α = 83.041 (14)°	*T* = 173 K
β = 82.868 (14)°	Plate, colourless
γ = 77.312 (14)°	0.34 × 0.23 × 0.12 mm
*V* = 562.80 (12) Å^3^	

### Data collection

**Table:**

Stoe IPDS diffractometer	2055 independent reflections
Radiation source: fine-focus sealed tube	1682 reflections with *I* > 2σ(*I*)
graphite	*R*_int_ = 0.056
φ scans	θ_max_ = 26.0°, θ_min_ = 2.7°
Absorption correction: multi-scan (MULscanABS in *PLATON*; Spek, 2009)	*h* = −7→7
*T*_min_ = 0.771, *T*_max_ = 1.353	*k* = −9→8
4473 measured reflections	*l* = −14→13

### Refinement

**Table:**

Refinement on *F*^2^	Primary atom site location: structure-invariant direct methods
Least-squares matrix: full	Secondary atom site location: difference Fourier map
*R*[*F*^2^ > 2σ(*F*^2^)] = 0.044	Hydrogen site location: inferred from neighbouring sites
*wR*(*F*^2^) = 0.114	H-atom parameters constrained
*S* = 0.98	*w* = 1/[σ^2^(*F*_o_^2^) + (0.0795*P*)^2^] where *P* = (*F*_o_^2^ + 2*F*_c_^2^)/3
2055 reflections	(Δ/σ)_max_ < 0.001
136 parameters	Δρ_max_ = 1.49 e Å^−^^3^
0 restraints	Δρ_min_ = −1.27 e Å^−^^3^

### Special details

**Table:**

Geometry. Bond distances, angles *etc*. have been calculated using the rounded fractional coordinates. All su's are estimated from the variances of the (full) variance-covariance matrix. The cell e.s.d.'s are taken into account in the estimation of distances, angles and torsion angles
Refinement. The NH_2_H-atoms were included in calculated positions and treated as riding atoms: N—H 0.88 Å with *U*_iso_(H) = 1.2*U*_eq_(parent N-atom).

### Fractional atomic coordinates and isotropic or equivalent isotropic displacement parameters (Å^2^)

**Table:**

	*x*	*y*	*z*	*U*_iso_*/*U*_eq_	
Ag1	0.48799 (7)	0.87109 (6)	0.15613 (4)	0.0303 (2)	
Ag2	1.07497 (7)	1.16730 (7)	0.06513 (4)	0.0327 (2)	
Cl1	0.6808 (2)	1.16001 (19)	0.05756 (13)	0.0245 (4)	
S1	0.7764 (2)	0.63258 (18)	0.08944 (12)	0.0197 (4)	
S2	0.2032 (2)	1.07487 (18)	0.25782 (13)	0.0201 (4)	
N1	1.1617 (8)	0.5710 (7)	0.1682 (5)	0.0264 (16)	
N2	0.8858 (7)	0.5149 (7)	0.2959 (5)	0.0271 (16)	
N3	0.0979 (8)	0.8245 (7)	0.4138 (5)	0.0319 (16)	
N4	−0.1790 (8)	0.9882 (7)	0.3218 (5)	0.0276 (16)	
C1	0.9560 (8)	0.5703 (7)	0.1930 (5)	0.0179 (14)	
C2	0.0266 (8)	0.9512 (7)	0.3359 (5)	0.0183 (17)	
O1	0.1853 (6)	0.3896 (6)	0.4594 (4)	0.0303 (15)	
O2	0.4735 (7)	0.2157 (7)	0.5106 (5)	0.0440 (16)	
O3	0.4800 (8)	0.3913 (8)	0.3521 (5)	0.0481 (18)	
N5	0.3826 (7)	0.3331 (7)	0.4412 (5)	0.0268 (16)	
H1A	1.25110	0.53410	0.22110	0.0320*	
H1B	1.21010	0.60840	0.09880	0.0320*	
H2A	0.97600	0.47810	0.34840	0.0330*	
H2B	0.74820	0.51410	0.31310	0.0330*	
H3A	0.00830	0.76530	0.45590	0.0380*	
H3B	0.23560	0.79830	0.42420	0.0380*	
H4A	−0.26680	0.92790	0.36460	0.0330*	
H4B	−0.22930	1.07330	0.26960	0.0330*	

### Atomic displacement parameters (Å^2^)

**Table:**

	*U*^11^	*U*^22^	*U*^33^	*U*^12^	*U*^13^	*U*^23^
Ag1	0.0177 (3)	0.0356 (3)	0.0341 (3)	−0.0027 (2)	0.0032 (2)	−0.0001 (2)
Ag2	0.0266 (3)	0.0465 (3)	0.0235 (3)	−0.0128 (2)	−0.0058 (2)	0.0155 (2)
Cl1	0.0214 (6)	0.0313 (7)	0.0219 (8)	−0.0123 (5)	−0.0056 (5)	0.0089 (6)
S1	0.0155 (6)	0.0263 (7)	0.0163 (8)	−0.0040 (5)	−0.0036 (5)	0.0038 (6)
S2	0.0160 (6)	0.0234 (7)	0.0198 (8)	−0.0062 (5)	−0.0013 (5)	0.0057 (6)
N1	0.024 (2)	0.036 (3)	0.019 (3)	−0.010 (2)	−0.0043 (19)	0.007 (2)
N2	0.014 (2)	0.043 (3)	0.022 (3)	−0.007 (2)	−0.0018 (19)	0.008 (2)
N3	0.019 (2)	0.042 (3)	0.029 (3)	−0.005 (2)	−0.005 (2)	0.019 (3)
N4	0.018 (2)	0.041 (3)	0.022 (3)	−0.011 (2)	−0.0029 (19)	0.014 (2)
C1	0.018 (2)	0.016 (2)	0.019 (3)	−0.0056 (19)	−0.002 (2)	0.005 (2)
C2	0.019 (3)	0.022 (3)	0.012 (3)	−0.001 (2)	−0.001 (2)	−0.001 (2)
O1	0.0158 (18)	0.044 (3)	0.024 (3)	0.0012 (17)	−0.0020 (16)	0.011 (2)
O2	0.022 (2)	0.056 (3)	0.047 (3)	−0.001 (2)	−0.013 (2)	0.019 (3)
O3	0.028 (2)	0.071 (4)	0.041 (3)	−0.020 (2)	0.010 (2)	0.016 (3)
N5	0.016 (2)	0.035 (3)	0.028 (3)	−0.008 (2)	−0.002 (2)	0.007 (2)

### Geometric parameters (Å, °)

**Table:**

Ag1—Cl1	2.8393 (15)	N1—C1	1.314 (8)
Ag1—S1	2.4305 (15)	N2—C1	1.301 (8)
Ag1—S2	2.4278 (15)	N3—C2	1.305 (8)
Ag1—Cl1^i^	2.9280 (16)	N4—C2	1.311 (8)
Ag2—Cl1	2.5477 (14)	N1—H1A	0.8800
Ag2—S2^ii^	2.4913 (16)	N1—H1B	0.8800
Ag2—S1^iii^	2.4827 (15)	N2—H2B	0.8800
S1—C1	1.738 (6)	N2—H2A	0.8800
S2—C2	1.744 (6)	N3—H3A	0.8800
O1—N5	1.242 (6)	N3—H3B	0.8800
O2—N5	1.241 (8)	N4—H4A	0.8800
O3—N5	1.244 (8)	N4—H4B	0.8800
			
Cl1—Ag1—S1	96.91 (5)	C1—N1—H1A	120.00
Cl1—Ag1—S2	90.60 (5)	C1—N2—H2A	120.00
Cl1—Ag1—Cl1^i^	92.64 (5)	H2A—N2—H2B	120.00
S1—Ag1—S2	169.00 (5)	C1—N2—H2B	120.00
Cl1^i^—Ag1—S1	82.74 (5)	C2—N3—H3A	120.00
Cl1^i^—Ag1—S2	105.01 (5)	H3A—N3—H3B	120.00
Cl1—Ag2—S2^ii^	114.00 (5)	C2—N3—H3B	120.00
Cl1—Ag2—S1^iii^	114.55 (5)	C2—N4—H4B	120.00
S1^iii^—Ag2—S2^ii^	126.77 (5)	H4A—N4—H4B	120.00
Ag1—Cl1—Ag2	123.99 (6)	C2—N4—H4A	120.00
Ag1—Cl1—Ag1^i^	87.36 (4)	O2—N5—O3	122.5 (5)
Ag1^i^—Cl1—Ag2	121.83 (6)	O1—N5—O2	118.6 (5)
Ag1—S1—C1	108.15 (19)	O1—N5—O3	118.8 (5)
Ag1—S1—Ag2^iii^	93.38 (5)	S1—C1—N1	121.4 (5)
Ag2^iii^—S1—C1	108.74 (19)	S1—C1—N2	119.0 (4)
Ag1—S2—C2	108.27 (19)	N1—C1—N2	119.6 (5)
Ag1—S2—Ag2^iv^	83.91 (5)	S2—C2—N3	119.6 (4)
Ag2^iv^—S2—C2	107.4 (2)	S2—C2—N4	121.3 (4)
C1—N1—H1B	120.00	N3—C2—N4	119.0 (5)
H1A—N1—H1B	120.00		
			
S1—Ag1—Cl1—Ag2	−44.29 (8)	S2—Ag1—Cl1^i^—Ag1^i^	91.33 (5)
S1—Ag1—Cl1—Ag1^i^	83.00 (5)	S2—Ag1—Cl1^i^—Ag2^i^	−37.73 (8)
S2—Ag1—Cl1—Ag2	127.66 (7)	S2^ii^—Ag2—Cl1—Ag1	−46.44 (8)
S2—Ag1—Cl1—Ag1^i^	−105.06 (5)	S2^ii^—Ag2—Cl1—Ag1^i^	−157.13 (6)
Cl1^i^—Ag1—Cl1—Ag2	−127.29 (7)	S1^iii^—Ag2—Cl1—Ag1	156.10 (6)
Cl1^i^—Ag1—Cl1—Ag1^i^	−0.02 (9)	S1^iii^—Ag2—Cl1—Ag1^i^	45.41 (8)
Cl1—Ag1—S1—C1	90.6 (2)	Cl1—Ag2—S2^ii^—Ag1^ii^	127.70 (5)
Cl1—Ag1—S1—Ag2^iii^	−20.40 (5)	Cl1—Ag2—S2^ii^—C2^ii^	20.4 (2)
Cl1^i^—Ag1—S1—C1	−177.6 (2)	Cl1—Ag2—S1^iii^—Ag1^iii^	−123.22 (5)
Cl1^i^—Ag1—S1—Ag2^iii^	71.40 (5)	Cl1—Ag2—S1^iii^—C1^iii^	−12.8 (2)
Cl1—Ag1—S2—C2	179.2 (2)	Ag1—S1—C1—N1	−124.7 (4)
Cl1—Ag1—S2—Ag2^iv^	72.83 (5)	Ag1—S1—C1—N2	58.3 (5)
Cl1^i^—Ag1—S2—C2	86.3 (2)	Ag2^iii^—S1—C1—N1	−24.5 (5)
Cl1^i^—Ag1—S2—Ag2^iv^	−20.06 (5)	Ag2^iii^—S1—C1—N2	158.4 (4)
Cl1—Ag1—Cl1^i^—Ag1^i^	0.02 (10)	Ag1—S2—C2—N3	60.2 (5)
Cl1—Ag1—Cl1^i^—Ag2^i^	−129.06 (7)	Ag1—S2—C2—N4	−123.1 (5)
S1—Ag1—Cl1^i^—Ag1^i^	−96.64 (5)	Ag2^iv^—S2—C2—N3	149.4 (4)
S1—Ag1—Cl1^i^—Ag2^i^	134.30 (7)	Ag2^iv^—S2—C2—N4	−33.8 (5)

Symmetry codes: (i) −*x*+1, −*y*+2, −*z*; (ii) *x*+1, *y*, *z*; (iii) −*x*+2, −*y*+2, −*z*; (iv) *x*−1, *y*, *z*.

### Hydrogen-bond geometry (Å, °)

**Table:**

*D*—H···*A*	*D*—H	H···*A*	*D*···*A*	*D*—H···*A*
N1—H1A···O3^ii^	0.88	2.28	3.153 (8)	170
N2—H2A···O1^ii^	0.88	1.95	2.831 (7)	177
N2—H2B···O3	0.88	2.11	2.932 (7)	155
N3—H3A···O1^v^	0.88	2.00	2.881 (7)	174
N3—H3B···O2^vi^	0.88	2.08	2.930 (7)	163
N4—H4A···O2^v^	0.88	2.22	3.095 (8)	173
N1—H1B···Cl1^iii^	0.88	2.56	3.372 (6)	155
N4—H4B···Cl1^iv^	0.88	2.62	3.396 (6)	147

Symmetry codes: (ii) *x*+1, *y*, *z*; (v) −*x*, −*y*+1, −*z*+1; (vi) −*x*+1, −*y*+1, −*z*+1; (iii) −*x*+2, −*y*+2, −*z*; (iv) *x*−1, *y*, *z*.

## References

[bb1] Akrivos, P. D. (2001). *Coord. Chem. Rev.* **213**, 181–210.

[bb2] Ashraf, W., Ahmad, S. & Isab, A. A. (2004). *Transition Met. Chem.* **29**, 400–404.

[bb3] Aslandis, P., Divanidis, S., Cox, P. J. & Karagiannidis, P. (2005). *Polyhedron*, **24**, 853–863.

[bb4] Casas, J. S., Martinez, E. G., Sánchez, A., González, A. S., Sordo, J., Casellato, U. & Graziani, R. (1996). *Inorg. Chim. Acta*, **241**, 117–123.

[bb5] Hanif, M., Ahmad, S., Altaf, M. & Stoeckli-Evans, H. (2007). *Acta Cryst.* E**63**, m2594.

[bb6] Isab, A. A., Ahmad, S. & Arab, M. (2002). *Polyhedron*, **21**, 1267–1271.

[bb7] Macrae, C. F., Edgington, P. R., McCabe, P., Pidcock, E., Shields, G. P., Taylor, R., Towler, M. & van de Streek, J. (2006). *J. Appl. Cryst.* **39**, 453–457.

[bb8] Nomiya, K., Takahashi, S. & Noguchi, R. (2000). *J. Chem. Soc. Dalton Trans.* pp. 2091–2098.

[bb9] Pakawatchai, C., Sivakumar, K. & Fun, H.-K. (1996). *Acta Cryst.* C**52**, 1954–1957.

[bb10] Raper, E. S. (1996). *Coord. Chem. Rev.* **153**, 199–255.

[bb11] Sheldrick, G. M. (2008). *Acta Cryst.* A**64**, 112–122. 10.1107/S0108767307043930 18156677

[bb12] Spek, A. L. (2009). *Acta Cryst.* D**65**, 148–155. 10.1107/S090744490804362X PMC2631630 19171970

[bb13] Stocker, F. B., Britton, D. & Young, V. G. Jr (2000). *Inorg. Chem.* **39**, 3479–3483. 10.1021/ic990859l 11196805

[bb14] Stoe & Cie (2004). *EXPOSE*, *CELL* and *INTEGRATE* in *IPDSI Software* Stoe & Cie GmbH, Darmstadt, Germany.

[bb15] Tsyba, I., Mui, B.-K., Bau, R., Noguchi, R. & Nomiya, K. (2003). *Inorg. Chem.* **42**, 8028–8032. 10.1021/ic030149m 14632522

[bb16] Udupa, R. M., Henke, G. & Krebs, B. (1976). *Inorg. Chim. Acta*, **18**, 173–177.

[bb17] Zachariadis, P. C., Hadjikakou, S. K., Hadjiliadis, N., Michaelides, A., Skoulika, S., Ming, Y. & Xiaolin, Y. (2003). *Inorg. Chim. Acta*, **343**, 361–365.

